# SARS-CoV-2: tracing the origin, tracking the evolution

**DOI:** 10.1186/s12920-022-01208-w

**Published:** 2022-03-18

**Authors:** Konstantinos Voskarides

**Affiliations:** Department of Basic and Clinical Sciences, University of Nicosia Medical School, Nicosia, Cyprus

**Keywords:** COVID-19, Mutation, Fitness, Gene, Infection, MRCA, Molecular evolution

## Abstract

The origin of SARS-CoV-2 is uncertain. Findings support a “bat origin” but results are not highly convincing. Studies found evidence that SARS-CoV-2 was around for many years before the pandemic outbreak. Evidence has been published that the progenitor of SARS-CoV-2 already had the capability to bind strongly to the human *ACE2* receptor. This may be an indication that many other animal viruses are capable to jump to humans, having already affinity for a human receptor. This is quite worrying since current ecosystems’ collapse brings people to high proximity with animals, increasing probabilities for random viral transitions. On the other hand, future adaptation of SARS-CoV-2 is of great concern. Virus-host interactions are complicated and unfortunately, we still do not have accurate tools for predicting viruses’ future evolution. Viral adaptation is a multifactorial process and probably SARS-CoV-2 will not become soon, as we wish, a harmless infection. However, humanity is currently under the largest vaccination program and it’s of great interest to see if vaccinations will change the evolutionary game against the virus.

## Background

COVID-19 is the greatest pandemic of the last 100 years, with millions of people having died (https://covid19.who.int). On 1st of November 2021, the global death toll from the COVID-19 pandemic passed 5 million [1]. This is far away from the concept of “common cold”. Despite the highly advanced genomic technology being available today, we are still not sure about the origin of SARS-CoV-2. A synthetic origin is doubted by many scientists [2, 3], arguing mainly on the similarity of the Receptor Binding Domain (RBD) with that of other coronaviruses. At present, 7.7 million SARS-CoV-2 genomes are presently registered in the GISAID database (https://www.gisaid.org). Tools, like Nextstrain [4], can use the registered genomes for phylogeographic analysis, permitting fast identification of the origin of new variants/mutations of the virus. In this perspective mini-review, available information about the origin of SARS-CoV-2 will be analysed, commenting also on the possible scenarios for the future adaptation of this virus in human populations. Examples of other pandemic viruses will be discussed, probably giving as some clues for the evolution of COVID-19.

## Main text

### Origin of SARS-CoV-2

Bats are a major reservoir for coronaviruses. RaTG13 was initially considered as the closest “relative” of SARS-CoV-2 [5, 6], a coronavirus that is found in *Rhinolophus* bats in China. Genomic similarity between the two viruses is 96% [5]. However, similarity in the RBD between SARS-CoV-2 and RaTG13 is below 90%, making unclear the close phylogenetic relationship between the two viruses [7]. Studies that followed found evidence for other bat circulating coronaviruses, more closely related to SARS-CoV-2 [6, 8–11]. RBD is the domain of the viral Spike protein that binds human *ACE2* protein, and it is responsible for enabling entry into human cells. The SARS-CoV-2 Spike protein has greater affinity with the human ACE2 receptor than its SARS-CoV-1 homolog, explaining the greater SARS-CoV-2 infectivity [12]. An intermediate host has been proposed, since two pangolin coronaviruses share similarity with SARS-CoV-2, PCoV-GD (91.2% sequence similarity) isolated from pangolins imported from Guangdong, and PCoV-GX (85.4% sequence similarity) isolated from pangolins imported from Guangxi [13, 14]. The RBD region of PCoV-GD has 96.8% amino acid sequence similarity with the RBD of SARS-CoV-2 [7]. The two pangolin coronaviruses can infect human cell cultures, whereas RaTG13 cannot [7]. However, the whole genome similarity of the two pangolin coronaviruses with SARS-CoV-2 is low, making their relationship with SARS-CoV-2 unclear. *Rhinolophus* bats are considered the most probable origin of SARS-CoV-2 by many scientists [15].

Genome analysis of SARS-CoV-2 revealed multiple recombination events [16, 17]. When different viral strains infect the same host, genetic recombination is possible, creating new viral genomes. From this concept, hypothetically SARS-CoV-2 may be the result of pangolin and bat viruses’ recombination in a single host of unknown identity. SARS-CoV-2 recombination is a great concern for virologists since different viral variants can be combined into a more dangerous strain [16].

A critical question is: when did the SARS-CoV-2 Spike protein evolve its high affinity for human *ACE2* and did the recent ancestor already have this ability [6, 18]? Brintnell et al. 2021 [19], performed a detailed phylogenetic analysis, ancestral sequence reconstruction, and in situ molecular dynamics simulations to examine the SARS-CoV-2’s Spike-RBD’s functional evolution. They found astonishing evidence that the ancestor of RaTG13 and SARS-CoV-2 had a latent ability to bind strongly to the human *ACE2* receptor. The same team found that the high affinity of SARS-CoV-2 for human *ACE2* had been fully acquired about 7–50 years ago. In the same line with Brintnell et al. 2021, another team showed that SARS-CoV-2 had been evolved long before the pandemic emergence, few decades back (95% HPD: 1930–2000) [20], considering RaTG13 as the closest virus to SARS-CoV-2. Interestingly, the same team found that SARS-CoV-1 has similar divergence time with SARS-CoV-2, 40–70 years, using known extant bat virus lineages. Wang et al. [21], performed similar estimations, dating the most recent common ancestor (MRCA) of SARS-CoV-2 and RaTG13 to 51.71 years (95% CI, 28.11–75.31). Starr et al. [22], by using high-throughput assays, they analysed the evolutionary history of *ACE2* binding across a diverse range of sarbecoviruses. They found that this is an ancestral trait, and it is highly evolvable.

Taking into account the SARS-CoV-2 dating and its MRCA properties, three scenarios are most probable: (a) The SARS-CoV-2 ancestor has been incubating for years inside bats, accumulating mutations, and probably through a random event, e.g. in the Huanan wet market, the virus was transmitted in humans, (b) A less virulent SARS-CoV-2 ancestor was infecting humans for years, until accumulation of mutations increased its virulence, (c) The SARS-CoV-2 ancestor has been circulating in intermediate hosts until transmission to humans by a random event. Interestingly, Pekar et al. [23], using a coalescence approach, define the period between mid-October and mid-November 2019 as the possible period that the first case of SARS-CoV-2 emerged in Hubei province, China. Likewise, Xia [24], dated the common ancestor of sampled SARS-CoV-2 genomes to 16 August 2019 with a large tree of 83,688 genomes.

Ancestral capacity of animal viruses to bind human receptors is not an exclusivity of coronaviruses. Here, I would like to “borrow” the knowledge we gained from another pandemic of the previous century, HIV/AIDS. Despite the fact HIV is a very different virus from coronaviruses, it is similarly an animal-derived virus. There are two main HIV strains, HIV-1 and HIV-2, which are distantly related. They jumped from other primate species (SIV viruses) [25] to humans under independent transmission events [26]. Additionally, HIV-1 is not just one virus, but it represents four different groups, M, N, O, and P. Evidence shows that each group passed to humans by an independent cross-species transmission event [26]. These data show clearly that many different clusters of SIVs had the capability to infect humans, probably due to a latent property of their progenitors. Phylogenetic analysis of HIV-1 group M dated the most recent common ancestor to 1910–1930 [27], showing that the virus was circulating in humans long before the first documented case, like in case of SARS-CoV-2. The emergence of HIV-1 and HIV-2 as independent events can be compared with the emergence of SARS-CoV-1 and SARS-CoV-2. HIV-1 and HIV-2 both have a primate origin; SARS-CoV-1 and SARS-CoV-2 both have a bat origin. Environmental conditions of emergence are also comparable, this showing that viruses of the same species family can have similar emergence ways. HIV-1 and HIV-2 have passed to humans in African forests, probably by primate raw meat consumption. SARS-CoV-1 and SARS-CoV-2 have first passed to humans in a large city of China, probably in a wet market, directly or through an intermediate host. It would not be unlikely to have more future transition events of SARS-CoV-2 or its progenitor from animals to humans—like the case of HIV—especially if really the SARS-CoV-2 progenitor or its relatives (probably still existing) have the capability to infect humans.

### Evolution of SARS-CoV-2

It seems that coronavirus transitions to humans are not rare events. Besides SARS-CoV-1, SARS-CoV2 and MERS that cause severe infections, four more coronaviruses are known in humans, HCoV-229E, HCoV-OC43, HCoV-HKU1 and HCoV-NL63, that cause mild seasonal colds. SARS-CoV-1, SARS-CoV2 and MERS have recently jumped to humans, and therefore they have high virulence, despite having very different mortality rates, ~ 10%, < 1% and ~ 30% respectively [28]. Research has shown that after some time, viruses are adapted to hosts and by directional selection can become less harmful [28]. This time cannot be predicted.

Public media frequently write that SARS-CoV-2 will soon become a harmless virus, like the four known human coronaviruses causing seasonal colds. Unfortunately, viral adaptational process needs a lot of time. The MRCA dating of the four human coronaviruses causing mild infections is ranging from 150 to 800 years ago [29]. There is significant evidence that a “flu like” pandemic that killed about 1 million people between 1889 and 1891, has been caused by the HCoV-OC43 coronavirus, belonging in the four known mild human coronaviruses. The HCoV-OC43 dating is compatible with the date of this pandemic event, known as the “Russian flu” [30]. It is worth mentioning here that HCoV-OC43 is a Beta Coronavirus, like SARS-CoV-1, SARS-CoV-2, and MERS, but it belongs to a different subgenus (Embecovirus). SARS-CoV-1 and SARS-CoV-2 have a very different accessory ORF complement than the other human Coronaviruses [31]. This may possibly function as a barrier for single point recombination events between SARS-CoV-2 and the other circulating human coronaviruses. However, analysis by Nikolaidis et al. [31] shows that modular recombination of the Spike ORF between SARS-CoV-2 and the other human coronaviruses may theoretically be possible. This would be catastrophic, if such an event occurred between SARS-CoV-2 and MERS.

Many studies have already showed that SARS-CoV2 is possibly under strong purifying selection [29], meaning that most functional mutations are excluded from human populations. This is encouraging, but the problem is that the spread of SARS-CoV2 in human populations is huge. Making this clearer, if e.g. for every 100,000,000 mutations 99,999,999 disappear, but one with high transmissibility survives, then this is a problem. If this mutation is also of high virulence, then then problem is even bigger. Currently, a SARS-CoV-2 variant called Omicron, causing a milder disease, has highly replaced all the other variants of the virus in all over the world. The omicron variant’s spread raises doubts on SARS-CoV-2 purifying selection. Many people think that this may be the end of the pandemic since most of us we will finally get immunized by this variant. This is not a guaranteed scenario. Severe variants can still arise. Additionally, uncertainty exists since mutations of Omicron variant do not make sense when compared with the previous variants of the virus. The origin of this variant is still under investigation [32–34].

Readers may find interesting reading the paper by Amoutzias et al. [35], where five possible scenarios are analyzed for the future evolution of SARS-CoV-2. In brief: scenario 1: structural constraints limit any further evolution of the SARS-CoV-2 spike, scenario 2: new mutations or intra-SARS-CoV-2 recombination events lead to the evolution of novel SARS-CoV-2 strains, scenario 3: recombination events between SARS-CoV-2 and other sarbecoviruses, scenario 4: recombination events between SARS-CoV-2 and viruses from other Beta-CoV subgenera, scenario 5: non-homologous recombination of SARS-CoV-2 with other viruses.

Beyond directional selection, two more evolutionary processes are likely contributing to less harmful viral infections: (a) Viral strains that cause severe infections disappear, if their hosts eventually die or become socially restricted, and (b) People that died from SARS-CoV-2, vaccinated, or not, probably had certain HLA variant combinations that predisposed them for severe infection. These HLA combinations are probably gradually lost from human populations. Presently, this is not a proven evolutionary mechanism.

## Conclusions

There is increasing worry about the emergence of more pandemic agents in the future. Climate change and ecosystem collapse bring humans and animals in greater contact more frequently. Obviously, this can increase zoonotic outbreaks [36]. Many animal viruses with capability to infect humans are “waiting” for the chance to cross the species barrier. Obviously, we must upgrade zoonotic disease surveillance in all over the world. We must be prepared to anticipate or even better to prevent future pandemic outbreaks.

I am afraid that we must adjust our lives to COVID-19 for many years from now. The best-case scenario would probably be natural immunity, meaning that most people on Earth will be finally immunized by natural infection from SARS-CoV-2. This could be indeed the final point of this pandemic, but new viral strains can still arise, of unknown virulence, able to re-infect humans. Don’t forget that viruses like HIV, HBV, HBC and Ebola virus, infect humans for decades and still are too virulent. We are dealing with a huge viral spread. Currently the best we can do is to invest to vaccination strategies.

## Data Availability

Not applicable.

## Abbreviations

MRCA: Most recent common ancestor
RBD: Receptor binding domain
ORF: Open reading frame
